# Physico-chemical dataset from an *in situ* mesocosm experiment simulating extreme climate events in Lake Geneva (MESOLAC)

**DOI:** 10.1016/j.dib.2021.107150

**Published:** 2021-05-14

**Authors:** Viet Tran-Khac, Pascal Perney, Laura Crépin, Philippe Quetin, Isabelle Domaizon, Stéphan Jacquet, Laurent Espinat, Clémentine Gallot, Serena Rasconi

**Affiliations:** a Université Savoie Mont Blanc, INRAE, CARRTEL^1^, 74200 Thonon les Bains, France; b Laboratoire d'Océanologie et de Géosciences, UMR LOG 8187, 62930 Wimereux, France

**Keywords:** Physico-chemical dataset, Experimental ecology, Climate change, Large peri-alpine lakes

## Abstract

This dataset complement a previously published dataset [1] and corresponds to the physico-chemical parameters data series produced during the MESOLAC experimental project [2]. The presented dataset is composed of: 1. *In situ* profiles (0–3m) of temperature, conductivity, pH, dissolved oxygen (concentration and saturation). 2. *In situ* measurements of light spectral UV/VIS/IR irradiance (300–950 nm wavelength range) taken at 0, 0.25, 0.5, 1, 1.5, 2 and 2.5m. 3. Laboratory chemical analysis of samples collected at 0 and 2 m (conductivity, pH, total alkalinity, NH_4_, NO_2_, NO_3_, total and particulate nitrogen (Ntot, Npart), PO_4_, total and particulate phosphorus (Ptot, Ppart), total, organic particulate and total particulate carbon (Ctot, Cpart-org, Cpart-tot), Cl, SO_4_, SiO_2_. 4. Laboratory analysis of pigments extracted from samples collected at 0 and 2 m (Chl*a*, Chl*c*, carotenoids, phaeopigments).

The experimental design is the same as in Tran-Khac et al [1]. Briefly, it consisted of nine pelagic mesocosms (about 3000 L, 3m depth) deployed in July 2019 in Lake Geneva near the shore of Thonon les Bains (France) aiming to simulate predicted climate scenarios (i.e. extreme events) and assess the response of planktonic communities, ecosystem functioning and resilience.

During the experiment, physical parameters were measured twice a week. At the same time, samples were collected at 0 and 2m of depth for subsequent chemical laboratory analyses. These data are presented in the dataset file, ordered by sampling event (numbered from S1 to S8), treatment (Control-C, High-H and Medium-M) and replicates (1 to 3). For each sampling point the measured parameters are listed in columns, missing data and values below the detection limit are marked as NA (not available).

This data set aims to contribute to the understanding of the effect of environmental forcing on lake physico-chemical characteristics (such as temperature, oxygen and nutrient concentration) under simulated intense weather events. To a broader extent, the presented data can be used for a wide variety of applications, including monitoring of a large peri-alpine lake functioning under environmental stress and being included in further meta-analysis to generalise the effect of climate change on large lakes. The two complementary dataset differ in the acquired data and methods, temporal and spatial resolution. They complete each other in terms of physico-chemical characterization of the experimental treatments and together can allow comparison of the two different monitoring strategies (continuous vs punctual) during *in situ* experimental manipulations.

## Specifications Table

**Table:** 

Subject	Environmental Science - Ecology
Specific subject area	Physico-chemical dataset produced during an *in situ* mesocosm experiment in Lake Geneva.
Type of data	Tables
How data were acquired	Measured parameters, used devices and standard methods [3] for acquisition include: 1. *In situ* profiles (0–3m) of temperature, conductivity, pH, dissolved oxygen (concentration and saturation) acquired using the multiparameter probe YSI EXO1. 2. *In situ* measures of light spectral UV/VIS/IR irradiance (300–950 nm wavelength range) taken at 0, 0.25, 0.5, 1, 1.5, 2 and 2.5m using the RAMSES-ASC-VIS irradiance sensor. 3. Laboratory chemical analysis of samples collected at 0 and 2m of: ‐ Conductivity and pH measured using potentiometric and conductometric method (ISO 10523:2008). ‐ Total alkalinity measured by potentiometric determination (ISO 9963-1). ‐ NH_4_, NO_2_, NO_3_, total and particulate nitrogen (N total and N particulate) measured using an ElementarVario© TOC/TNb coupled with a chemiluminescence detector (APNA-370; Horiba©) after high temperature digestion and catalytic post combustion (ISO 5664:1984; ISO 6777:1984; ISO 10304- 1; EN 12260:2003; ASTM D2579-93e1).
	‐ PO_4_, total and particulate phosphorus (P tot and P part) measured using a UV-VIS spectrophotometer (Cary 50 scan; Varian©) following sulphuric acid digestion and a molybdenum blue method (EPA 365.3). ‐ Total, organic particulate and total particulate carbon (C tot, Corg part and Cpart) using a CHN elemental analyser following high-temperature combustion method with a gas chromatograph equipped with a thermal conductivity detector (Flash 2000; Thermoscientific©) (ASTM D2579-93e1). ‐ Cl^−^ and SO_4_^−^, quantified using ion exchange (861 Advanced Compact ion chromatograph; Metrohm©) with chemical suppression (ISO 10304-1). ‐ SiO_2_, analysed using a Smartchem 200 Discrete Analyzer (WESTCO Scientific Instruments©) (NF T90-007). 4. Chl*a*, Chl*c*, carotenoids and pheopigments were analysed using spectrophotometry after extraction into 90% acetone (ISO 10260: 1992).
Data format	Raw
Parameters for data collection	Data were acquired twice a week during the four weeks of the experiment (July 4 – 30, 2019). *In situ* profiles were acquired in every mesocosm from the surface (0m) to the bottom of the mesocosms (3m) and in the lake for comparison with natural conditions. Discrete samples were taken using a standard Niskin bottle at the same date at 0 and 2 meters of depth for laboratory analyses.
Description of data collection	This dataset was produced during the same experiment of the dataset published in Tran-Khac et al 2020 [1]. The data collection is different in the temporal and spatial resolution. The data in the present dataset were acquired during samplings every 2-4 days, while in the previous dataset data were produced by automated loggers performing continuous data acquisition every 15 minutes. The spatial resolution is different as in the present dataset we present whole mesocosms depth profiles from multiparametric probe and samples taken at 0 and 2m for measures using standard lab methods. The loggers used for data acquisition in the previous dataset were placed at the fixed depths of 0.15, 0.25, 1 and 2m.
Data source location	Institution: UMR INRAE CARRTEL City/Town/Region: Thonon les Bains Country: France The mesocosms were placed in a rectangle with coordinates: 46°22′09.64′′ N 6°27′09.89′′ E 46°22′11.39′′ N 6°27′08.73′′ E 46°22′ 12.58′′ N 6°27′ 13.74′′ E 46°22′ 11.19′′ N 6°27′ 14.80′′ E
Data accessibility	The dataset described in this data paper is archived with the manuscript as Supporting Information (Mesolac-Dataset_PhysChem.xlsx) and is accessible as open file in the INRAE Dataverse repository as single excel file [2]. Repository name: Dataverse INRAE Data identification number: https://doi.org/10.15454/PCOPYW Direct URL to data: https://data.inrae.fr/dataset.xhtml?persistentId=doi:10.15454/PCOPYW
Related research article	Complementary DIB article: [1] V. Tran-Khac, P. Quetin, I. Domaizon, S. Jacquet, L. Espinat, C. Gallot, S. Rasconi. In situ pelagic dataset from continuous monitoring: a mesocosm experiment in Lake Geneva (MESOLAC) (2020). Data in Brief. 32:106255. https://doi.org/10.1016/j.dib.2020.106255 The complementary dataset published in Tran-Khac et al 2020 produced during the same experiment is available at: https://data.inra.fr/dataset.xhtml?persistentId=doi:10.15454/T3VCB0

## Value of the Data

• This data set improves our understanding of the effect of environmental forcing on lake physico-chemical characteristics (such as temperature, oxygen and nutrient concentration) under simulated intense weather events.
• These data provide a quality-assured baseline of key lake parameters measured using standardised methods that may be used to discern changes in lake environmental status and assist in better managing and protecting lake ecosystems.
• The two complementary datasets complete each other in terms of physico-chemical characterization of the experimental treatments. They differ in the acquired data and methods, temporal and spatial resolution. Together they can allow comparison of the two different monitoring strategies (continuous vs punctual) during *in situ* experimental manipulations.

## 1 Data Description

The data are stored as single excel file containing four sheets [2]. The first sheet (named “Physical”) contains *in situ* profiles (0–3m) of temperature, conductivity, pH, oxygen saturation and oxygen concentration. The second sheet (named “Light”) contains *in situ* measures of light spectral UV/PAR/IR irradiance (300–950 nm wavelength range) taken at 0, 0.25, 0.5, 1, 1.5, 2 and 2.5m. The third sheet (named “Chemical”) contains data from laboratory chemical analysis of samples collected at 0 and 2m (conductivity, pH, total alkalinity, NH_4_, NO_2_, NO_3_, Ntot, Npart, PO_4_, Ptot, Ppart, Ctot, Cpart-org, Cpart-tot, Cl, SO_4_, SiO_2_). Samples labelled with “A” in the column “Sample_Name” are the TOC measure taken at the surface of the enriched mesocosms immediately after the DOC solution was added. The fourth sheet (named “Pigments”) contains data from laboratory analysis of pigments extracted from samples collected at 0 and 2m (Chl*a*, Chl*c*, carotenoids, pheopigments). The measured parameters are listed in columns, including the concentration measure unit (row flagged by “#”). Unique ID for each column (Sample_Name) includes the mesocosm treatment and replicate, the sampling event and the depth of the measured parameter for the discrete samples taken at 0 and 2m. Missing data and values below the detection limit are identified by the code NA.

## 2 Experimental Design, Materials and Methods

**Experimental design:** The mesocosms consisted of reinforced polyethylene bags (produced by Insinööritoimisto Haikonen Oy, Finland), supported at every meter of depth by plastic frames to avoid collapse of the structure due to the lake currents and supported by a double system of buoys at the surface to allow floating. Each bag was filled with water the same day within a few hours and the mesocosms were left to acclimate for three days before the start of the experiment. The experiment was performed in Lake Geneva, from 4th to 30th July 2019, with the aim to simulate predicted climate scenarios in a deep peri-alpine lake during a period of high production. The experimental design consisted of nine pelagic mesocosms (about 3000 L, 3m depth) placed in the lake epilimnion near the shore of Thonon les Bains, France. The experiment included three treatments each replicated three times: a control (no treatment applied – named C) and two different treatments simulating medium and high intensity extreme weather events. The high intensity treatment (named H) aimed at reproducing short and intense weather events such as violent storms. It consisted of a short-term intense stress applied for five days during the first week (from July 4 to 8), with high pulses of dissolved organic carbon (5x increased concentration compared to the control, i.e. total DOC ~ 6 mgL^−1^), transmitted light reduced to 15% and water column manual mixing applied daily for 15 minutes. The medium intensity treatment (named M) simulated less intense and more prolonged exposures such as flood events. It was maintained for four weeks and consisted of 1.5x increased concentration of dissolved organic carbon (i.e. total DOC ~ 2 mgL^−1^), 70% transmitted light and water column manual mixing applied daily for 5 minutes. To reduce the transmitted light we used LEE ND (Neutral Density) filters, which reduce light without changing colour. To expose all the mesocosms to the same covering condition the control treatments were covered with a 95% transmitted light filter. The manual mixing was done by lowering and lifting in the mesocosms a disc of 0.5m diameter with holes to allow the water to flow trough.

The experimental design is the same as described in the primary dataset [1].

**Instrumentation:** Physico-chemical characterisation of each mesocosm included *in situ* profiles of temperature, conductivity, pH, dissolved oxygen (concentration and saturation) acquired using a multiparameter probe YSI EXO1. Light spectral measurements of UV/VIS/IR irradiance (300-950 nm wavelength range) were taken at 0, 0.25, 0.5, 1, 1.5, 2 and 2.5m using a RAMSES-ASC-VIS irradiance sensor. Laboratory analyses of chemical parameters were performed following standard methods as described in [3] and using the instruments listed in the Specifications Table.

**Data forms or acquisition methods**: *In situ* profiles and discrete samples for data analyses were collected twice a week during the experiment. Data from the automated probes are provided in the form of csv or txt files and were downloaded after each sampling event. Water samples for chemical analyses were stored at 4°C and the analyses were performed within 48 h after collection.

**Data entry verification procedures**: *In situ* data were collected and visually checked by the operator after each time of conducting the probes. They were cross-checked by a second operator using the original field data sheets. Laboratory analysis data were collected and manually checked by the operator and validated by authorized scientific staff.

**Quality assurance/quality control procedures**: *In-situ* data were manually validated and cross-validated with laboratory analysis (pH, Cond, dissolved oxygen).

The analytical quality control of laboratory data followed a rigorous traceable workflow from sample collection to data validation. Data were controlled by an analytical quality monitoring of instruments, verification with reference materials and manual cross-validation between chemist staff.

**Data anomalies**: Negative values of light spectral measurement were detected near 300 nm wavelengths. According to manufacturer’ specifications, negative values can be included in uncertainty interval and were marked as NA.

**Computer programs and data-processing algorithms**: For data formatting, homogenization and first visualization we used the software R. Data outliers were mostly manually identified based on quality assurance and quality control procedures (previous section).

## Declaration of Competing Interest

The authors declare that they have no known competing financial interests or personal relationships, which have, or could be perceived to have, influenced the work reported in this article.

## Appendix Supplementary materials

Image, application 1
